# Endophytic fungus *Colletotrichum* sp. AP12 promotes growth physiology and andrographolide biosynthesis in *Andrographis paniculata* (Burm. f.) Nees

**DOI:** 10.3389/fpls.2023.1166803

**Published:** 2023-07-04

**Authors:** Dan Xu, Na Li, Yuan-Qin Gu, Jin Huang, Bin-Sheng Hu, Jian-Yun Zheng, Jing-Wen Hu, Qin Du

**Affiliations:** Medicinal Plant Biotechnology Laboratory, College of Chinese medicine, Guangzhou University of Chinese Medicine, Guangzhou, China

**Keywords:** *Andrographis paniculata* (Burm. f.) Nees, *Colletotrichum* sp. AP12, elicitor components, andrographolide biosynthesis and accumulation, defense enzyme activity, plant growth

## Abstract

Endophytic fungi can promote host plant growth, enhance antioxidant defense enzyme activity, and induce the biosynthesis and accumulation of secondarymetabolites. Therefore, using endophytic fungi to improve the quality and yield of medicinal plants or important crops is an effective means of regulation. *Colletotrichum* sp. AP12 has been reported to produce andrographolide compounds (ADCs). This study aimed to investigate the effects of AP12 and its elicitors on the growth, defense enzyme activity, accumulation, and transcription levels of key genes in *Andrographis paniculata* (Burm. f.) Nees (*A. paniculata*). Using fermentation method to prepare AP12 into the inactivated fermentation solution (IFS), fermentation solution (FS), inactivated mycelium solution (IMS), and mycelium solution (MS), and the results showed that all four fungal elicitor components (ECs) could promote *A. paniculata* growth, enhance antioxidant defense enzymes, and increase ADC content and yield, especially the IMS group that had the highest leaf area, whole plant dry weight, superoxide dismutase (SOD), catalase (CAT) enzyme activities, total lactone contents, and yields, which were 2.37-, 1.60-, 2.20-, 3.27-, 1.59-, and 2.65-fold of the control, respectively. The 14-deoxyandrographolide (NAD) in the host irrigated with MS was 3.35-fold that of the control. In addition, AP12-infected *A. paniculata* sterile seedlings could significantly increase ADC content and expression levels of key enzyme genes, especially on day 12, when the total lactone content of the host reached 88.881± 5.793 mg/g DW, while on day 6, CPS gene expression level reached 10.79-fold that of the control, in turn promoting the biosynthesis and accumulation of andrographolide. In conclusion, the endophytic fungus AP12 is beneficial to the growth and secondary metabolism of *A. paniculata*, which is helpful for the cultivation and application of the biological bacterial fertilizer in *A. paniculata*, providing a theoretical and research basis for the use of endophytic fungi as a microbial resource to improve the quality and yield of medicinal plants.

## 1 Introduction

**Figure 1:**
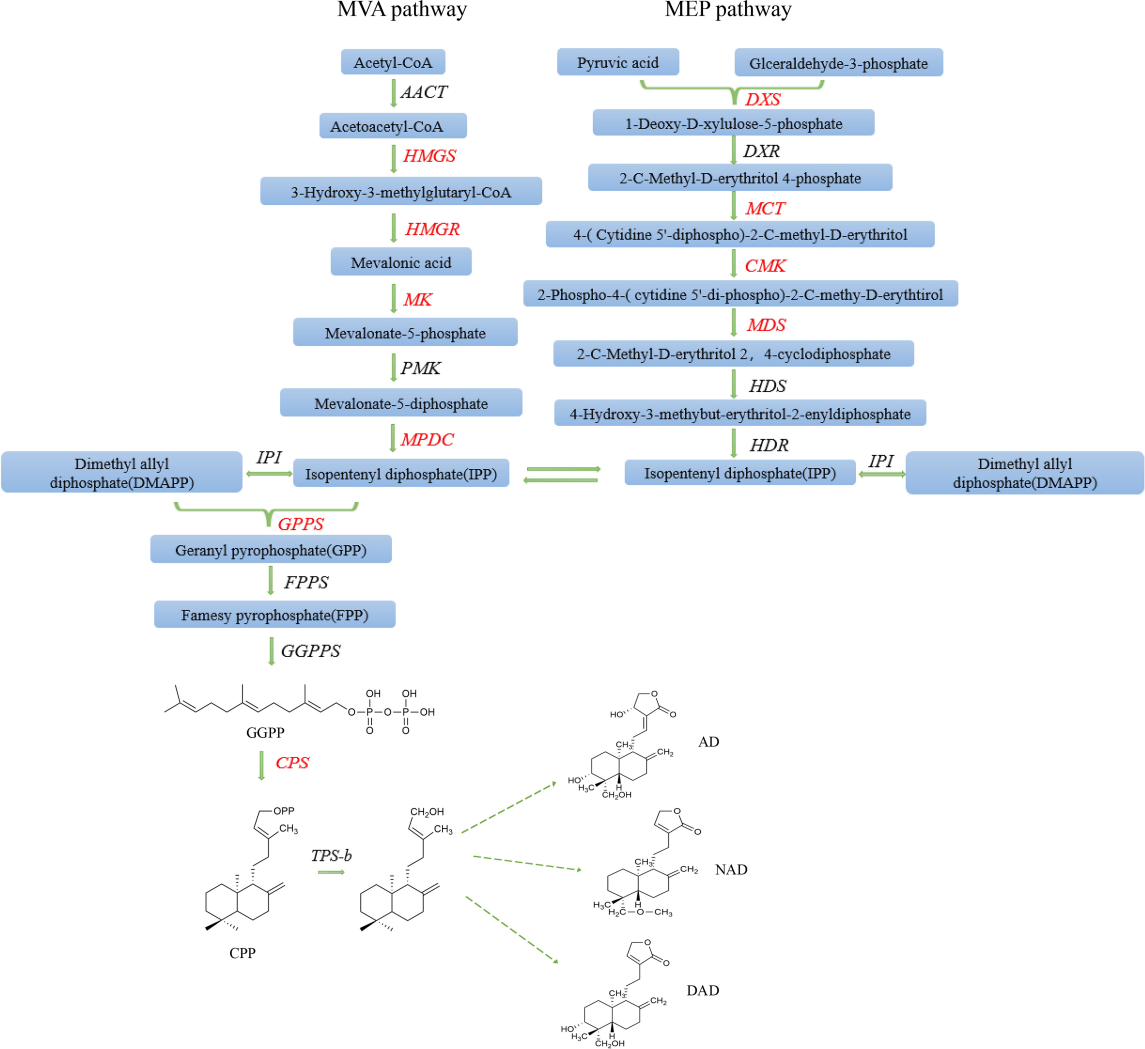
The MVA and MEP pathways involved in andrographolide biosynthesis.

*Andrographis paniculata* (Burm. f.) Nees (*A*. *paniculata*) is an herbaceous plant belonging to the genus Andrographis (Acanthaceae family) and is widely known as the “King of the bitters” or “Kalmegh” (Garg et al., 2015; Jiang et al., 2021). It is typically found in tropical and subtropical areas and has long been used as a traditional medicine in China, India, Malaysia, and other nations (Mishra et al., 2007). *A*. *paniculata* has been recorded in the Chinese Pharmacopoeia, Indian Pharmacopoeia, and British Pharmacopoeia and has been used as a dietary supplement according to the American Pharmacopoeia (Hossain et al., 2021). In addition, *A*. *paniculata* has been included in the World Health Organization (WHO) monographs on selected medicinal plants (World Health Organization, 2002). According to the literature, *A*. *paniculata* plants contain more than 20 diterpenoids and 10 flavonoids. The main active medicinal ingredients among these compounds are andrographolide compounds (ADCs), which belong to bicyclic diterpene lactones, a class of diterpenoids consisting of a five-membered lactone and a hemichrysane type of bicyclic skeletal structure (Srivastava and Akhila, 2010). Terpenoids are synthesized in the plant body mainly by two parent pathways together, the mevalonate pathway (MVA) in the cytoplasm and the 2-C-methyl-D-erythritol-4-phosphate pathway (MEP) in the plastid (Vranová et al., 2013). The MVA pathway uses acetyl-CoA as raw material, and the MEP pathway uses pyruvic acid and glyceraldehyde-3-phosphate as raw materials to synthesize isopentenyl diphosphate (IPP) and dimethyl allyl diphosphate (DMAPP) *via* a process mediated by a series of upstream rate-limiting enzymes (**Figure 1**) (Sun et al., 2019; Yao et al., 2020). Then, in the key link, the formation of various skeleton intermediates is catalyzed by terpene synthase, and they are modified downstream to form terpenoids (Wu et al., 2006). Diterpene compounds with a wide range of biological activities are commonly found in the plant world and are closely related to plant growth and development; for example, gibberellin is a plant growth regulator that promotes seed germination, stem elongation, and plant cell growth (Hedden, 2020). Diterpenoids are also used in medical research and have good therapeutic effects on certain major human diseases; for example, tanshinone is used to treat cardiovascular diseases, paclitaxel has antitumor effects, and triptolide has strong anti-inflammatory effects (Shen et al., 2016). Similarly, ADCs, including andrographolide (AD), neandrographolide (NAD), and 14-deoxyandrographolide (DAD), are mainly produced in the leaves and stems of *A*. *paniculata* (Lim et al., 2012). These active ingredients have universal pharmacological effects, such as anti-HIV, anti-inflammatory, and anti-fertility effects, and have been widely used to treat various inflammatory diseases in the clinic (Pholphana et al., 2013; Rafi et al., 2022). These diseases include diarrhea, fever, upper respiratory infections, other chronic diseases, and infectious diseases (Islam et al., 2018). Owing to the important medicinal therapeutic effects of ADCs, the market demand for *A*. *paniculata* is increasing annually. The National Medicinal Plant Board (NMPB) and the Indian Council of Forestry Research and Education (ICFRE) have conducted an extensive survey of the Indian herbal medicine market and estimated the comprehensive commercial demand of *A*. *paniculata* to be 2,000–5,000 metric tons (MT) (Gupta et al., 2019). However, the existence of many unfavorable factors, such as germplasm homogeneity, environmental damage, and crop rotation barriers, has resulted in genetic variation, segregation, degradation, mixed germplasms, and unstable contents and quality of the active medicinal components of *A*. *paniculata* (Valdiani et al., 2017). Therefore, there is an urgent need to breed good varieties of *A*. *paniculata* with high yields, stability, and contents of medicinal components.

Endophytic fungi exist in healthy plant roots, stems, leaves, etc., and do not cause the host plant to display overt signs of lesions at any point during its life cycle (Liu and Liu, 2018; Ancheeva et al., 2020). According to the fossil record (Rodriguez and Redman, 2008), endophytic fungi have been connected to plants for at least 400 million years, and they have developed a stable and advantageous associations with hosts over this protracted period of developmental evolution in response to changes in the host’s genes and environment (Wu et al., 2020). In this endophyte–host plant interaction, the host plants can supply the nutrients necessary for endophytic fungal growth, and the endophytic fungus induces a variety of growth physiological and biochemical responses in the host plant by secreting corresponding induced substances, which researchers refer to as biological elicitors or endophytic fungal elicitors (Zhao et al., 2005); these elicitors include the endophytic fungus, inactive mycelium or mycelium extracts, and strain fermentation solution etc. (Algar et al., 2012), which can promote host plant growth and development (Pappas et al., 2018), enhance disease resistance (Shrivastava et al., 2015), increase the accumulation of secondary metabolites (especially active ingredients) (Zhai et al., 2017), and produce the same or similar secondary metabolites as the host (Gomez and Hortolan Luiz, 2018). For instance, Ye et al. (2020) isolated *Chaetomium globosum* J162 and *Colletotrichum gloeosporioides* J211, which were reported to promote flavonoid, ginsenoside, and sugar accumulation in *goldenrod*. Based on the aforementioned impacts of endophytic fungi on medicinal plants, we assume that they are a significant biological element influencing the quality of medicinal plants, in addition to abiotic factors including the light source, carbon source, soil, and water (Wei and Jousset, 2017). No studies have investigated the effects of endophytic fungi and their elicitors on growth and andrographolide biosynthesis and accumulation in *A*. *paniculata*, so our study provides a theoretical basis for the future application of endophytic fungi to improve artificial cultivation techniques for *A. paniculata* and further improve its quality and yield.

In a previous study, an endophytic fungus isolated from *A*. *paniculata*, identified as *Colletotrichum* sp. AP12 (Li et al., 2022b), was shown to have a potent antioxidant capacity and antibacterial ability and produced ADCs in a rich medium. Nevertheless, the content of ADCs gradually decreased after repeated subculturing, and it was speculated that it was associated with symbiosis *A*. *paniculata*, resulting in the transfer or exchange of substances such as related enzymes or genes in the andrographolide biosynthesis pathway. Therefore, in this study, *Colletotrichum* sp. AP12, which can produce ADCs, was used to prepare four fungal elicitor components (ECs): inactivated fermentation solution (IFS), fermentation solution (FS), inactivated mycelium solution (IMS), and mycelium solution (MS). The analysis of the effect of the ECs on the growth, antioxidant defense enzyme activity, and ADC accumulation of *A*. *paniculata* under potted conditions and preliminary experimental results showed that the mycelium component was most strongly induced. Then, in terms of molecular regulation, the regulation of key gene transcription levels in the andrographolide biosynthesis pathway was further analyzed by infecting sterile *A*. *paniculata* seedlings with AP12 mycelium.

## 2 Materials and methods

### 2.1 Collection of plant and *Colletotrichum* sp. AP12 materials

Healthy samples of *A*. *paniculata* were collected from the “Yao-Wang-Shang” experimental field (23°5087.72600 N, 113°40042.72300E) of Guangzhou University of Chinese Medicine, Guangzhou City, Guangdong Province, China. The previously isolated and characterized endophytic fungal strain AP12 was cultured and maintained on potato dextrose agar (PDA) medium (200 g/L potato extract, 20 g/L sucrose, 14 g/L technical agar) and named *Colletotrichum* sp. AP12 (GenBank ID: OL477581).

### 2.2 Preparation of AP12 fungal elicitor components

A sterile hole punch with a 0.8-cm diameter was used to punch out eight samples of fungi, which were then placed in 400 ml of potato dextrose broth (PDB) medium (200 g/L potatoes, 20 g/L sucrose) and fermented and cultured for 10 days at 28°C, mixed at a speed of 150 rpm/min. The mycelium and the fermentation solution were separated by vacuum filtration. First, part of the fermentation solution (dilute 2-fold with sterile water) was inactivated in an autoclave at 121°C for 20 min to obtain IFS; another part was not inactivated to obtain FS. The mycelium was rinsed many times with sterile water, all the remaining water was absorbed with filter paper, and 2 g of mycelium was accurately weighed, fully ground in a mortar, placed in 200 ml of sterile water, ultrasonically treated for 30 min, mixed well to obtain MS, and inactivated in an autoclave at 121°C for 20 min to obtain IMS. The concentrations were evaluated *via* the phenol−sulfuric acid colorimetric method with glucose as the standard (Dubois et al., 1956). The above were all made into fungal ECs for subsequent use.

### 2.3 *A. paniculata* plant cultivation with AP12 fungal elicitor components

Peat soil:vermiculite:perlite at a ratio of 2:1:0.5 was mixed thoroughly and sterilized at 121°C for 1 h to produce sterile soil. *A. paniculata* seeds of plump grains of uniform quality were sown in sterilized soil, irrigation with the AP12 fungal ECs was performed once every 3 days with a volume of 20 ml each time, and the control was treated with the same amount of sterile water; three plants were treated per group, and the procedure was repeated nine times in each group. The plants were cultured under a 12-h/12-h photoperiod with 60% RH at a temperature of 26 ± 2°C. Nine plants were randomly selected from each treatment at 65 days; growth indicators (plant height, number of leaves, and leaf area), biomass (whole-plant fresh weight, aboveground part fresh weight, whole-plant dry weight, aboveground part dry weight), enzyme activities, ADC contents, and yields were examined; and measurements were repeated in triplicate for each indicator.

### 2.4 Determination of leaf antioxidant defense enzyme activity

#### 2.4.1 Superoxide dismutase enzyme activity

An SOD crude extract solution was obtained by adopting the water-soluble tetrazolium (WST-1) method (Peskin and Winterbourn, 2017). The leaves were ground into powder in liquid nitrogen, 0.1 g of the powder was weighed and added to a ninefold volume of phosphate buffer (0.1 mol/L pH 7–7.4), and the mixture was vortexed for 3 min and centrifuged at 5,000 rpm/min for 10 min at 4°C. The supernatant of each sample was diluted eightfold with phosphate buffer (0.1 mol/L pH 7–7.4). Then, 20 μl of crude extract solution and 200 μl of reaction solution were mixed thoroughly, and incubated at 37°C for 20 min. The control and the control blank received 20 μl of distilled water, and their absorbance at 450 nm was read in a microplate reader. When the SOD inhibition rate reached 50% in this reaction system, the corresponding amount of the enzyme was defined as one SOD activity unit (U).

#### 2.4.2 Catalase activity

To prepare the CAT crude extract solution, the ammonium molybdate colorimetric method was used (Slaughter and O’Brien, 2000). The leaves were ground into powder with liquid nitrogen, 0.1 g of the leaf powder was weighed, a ninefold volume of 0.9% saline was added, a 10% tissue homogenate was obtained under ice water bath conditions, and the homogenate was centrifuged at 2,500 rpm/min for 10 min at 4°C. Then, 50 μl of crude extract solution and 2.2 ml of reaction solution were mixed thoroughly, and in a 37°C water bath for accurate reaction for 1 min, a 200-μl aliquot was read at 405 nm with a microplate reader to obtain the absorbance value. In this reaction system, the amount of H_2_O_2_ decomposed by per gram of tissue was defined as one unit enzyme activity (U).

#### 2.4.3 Peroxidase activity

The POD crude extract solution was prepared and evaluated *via* the guaiacol method (Do et al., 2003). Leaves were ground into powder with liquid nitrogen, 0.1 g of leaves was weighed accurately, a ninefold volume of 0.1 mol/L pH 7–7.4 phosphate buffer was added, the mixture was ground in an ice water bath, and a 10% tissue homogenate was obtained and subjected to 3,500 rpm/min centrifugation at 4°C for 10 min. Then, 100 μl of crude extract solution and 2.9 ml of reaction solution were mixed thoroughly, and in a 37°C water bath for 30 min, and 1 ml of each reaction termination solution was combined and mixed well. The mixture was centrifuged at 3,500 rpm/min at 4°C for 10 min, and a 200-μl aliquot was read at 420 nm on a microplate reader to obtain the absorbance value. In this reaction, the amount of enzyme that catalyzed 1 μg of substrate per minute per gram of tissue was defined as one unit enzyme activity (U).

### 2.5 Determination of andrographolide compound content by HPLC

#### 2.5.1 Preparation of *A*. *paniculata* plant samples and andrographolide standard

Referring to the 2020 edition of the Chinese Pharmacopoeia (Chinese Pharmacopoeia Commission, 2020 edition), the samples of *A*. *paniculata* were cleaned and dried in an oven at 45°C to constant weight, and the ground powder was passed through the No. 4 sieve by the liquid nitrogen grinding method. Accurately weighed 0.100 ± 0.002 g of powder was soaked for 1 h with 40% MeOH in 5 ml, weighed and treated with an ultrasound (power 250 w, frequency 33 kHz) for 30 min, cooled, weighed again (40% methanol made up the lost weight), and shaken well *via* high-speed centrifugation at 10,000 rpm/min for 2 min at 4°C; the supernatant was taken and passed through a 0.22-μm microporous membrane to obtain 20 mg/ml of the test solution; AD standard (China Institute of Food and Drug Testing and Certification) was accurately weighed, methanol was added to make a solution containing 0.302 mg of AD standard per 1 ml and passed through a 0.22-µm microporous membrane, and then the standard solution was obtained. The peak areas of the AD standard were used as an internal reference and multiplied by the relative response factors (**Table 1**) to calculate the relative contents of AD, NAD, and DAD, according to the Chinese pharmacopeia.

**Table 1:** Relative retention time and HPLC correction factors of *A*. *paniculata* diterpene lactone.

Ingredients to be tested	Relative retention time	Correction factors
AD	1.00	1.00
NAD	1.95	1.12
DAD	2.18	0.79

#### 2.5.2 Determination of andrographolide, neandrographolide, and 14-deoxyandrographolide content

The contents of AD, NAD, and DAD were determined by HPLC using a HYPERBERE C18 column (250 mm × 4.6 mm, 5 μm, Shimadzu China Co., Ltd., Beijing) equipped with a dual-wavelength detector and workstation software. Approximately 5 µl of the samples of defined concentration were injected into the HPLC column and gradient elution using acetonitrile (A)–pure water (B): 0–15 min, 20%–25% A; 15–30 min, 25%–28% A; 30–60 min, 28%–40% A; 60–65 min, 40%–85% A. Detection wavelength: 205 nm, flow rate: 1 mg/ml.

### 2.6 Construction of the co-culture system of AP12-infected *A*. *paniculata* sterile seedlings

#### 2.6.1 Preparation and culture of *A*. *paniculata* sterile seedlings

*A*. *paniculata* seeds were first soaked in distilled water, vernalized at 4°C for 2 days, transferred to an ultraclean bench (Radobio, Shanghai, China), sterilized with 75% ethanol for 30 s and 12% sodium hypochlorite for 20 min, and rinsed in sterile water four times, after which sterile filter paper was used to absorb seed surface water. The seeds were transferred to culture bottles containing Murashige Skoog (MS) medium (Solarbio, Beijing, China) for culture, where MS medium was obtained by mixing 2.37 g/L MS powder, 15 g/L sucrose, and 6 g/L technical agar, adjusting the pH value to 6.0, and performing sterilization at 121°C for 20 min in an autoclave. Sterile *A. paniculata* seedlings were cultured under a 12-h/12-h photoperiod at 26±2°C and 60% RH.

#### 2.6.2 The *Colletotrichum* sp. AP12-infected *A. paniculata* sterile seedlings

The coculture system was constructed using the technique of grafting back to sterile seedlings (Leuchtmann and Clay, 1988). Two-month-old seedlings of the same size and morphology with four pairs of true leaves were selected as experimental materials, and AP12 mycelium was placed on the stem tip meristem of the seedlings with a 0.5-cm-diameter sterile inoculum ring on an ultraclean bench (Radobio, Shanghai, China), whereas the control group was left untreated. Fifteen plants were randomly selected from each group (totaling 180 plants) for each treatment from 0 to 15 days. Additionally, the sterile *A*. *paniculata* seedlings were cocultured with AP12 for 3, 6, 9, 12, and 15 days under the conditions of a 12-h/12-h photoperiod, 26±2°C temperature, and 60% RH, and the growth of *A*. *paniculata* was observed daily.

### 2.7 Immunocytochemical staining of the *Colletotrichum* sp. AP12-infected *A*. *paniculata* sterile seedlings

Immunocytochemical staining of plant tissue was performed as described (Nasr et al., 2006; Zhai et al., 2018). Samples of stem sections (0.5 cm) were collected near the middle of the sterile seedling and fixed with 4% paraformaldehyde for more than 24 h. Then, paraffin sections of 6 μm were prepared according to the following procedures. The slices were sequentially placed into dimethylbenzene xylene I for 15 min, dimethyl benzene xylene II for 15 min, anhydrous ethanol I for 8 min, anhydrous ethanol II for 8 min, 95% alcohol for 5 min, 90% alcohol for 5 min, 80% alcohol for 5 min, 75% alcohol for 5 min, and distilled water for rinsing. Then, the samples were placed in a retrieval box (Servicebio, Wuhan, China) filled with EDTA antigen retrieval buffer (pH 8.0) and boiled in a microwave oven for 5 min for antigen retrieval. After natural cooling, the slides were placed in PBS, pH 7.4, and rinsed three times (5 min each) on a decoloring table. Then, the slices were spin-dried, and a Pap Pen (Servicebio, Wuhan, China) was used to draw a circle in the center of the slices to prevent the flow of antibodies. Next, 3% BSA was dropped into the circle to cover the tissue evenly, and the sample was stored at room temperature for 30 min. After gently shaking off the blocking solution, the primary antibody labeled with FITC (1:100 concanavalin A; GenBank ID: 72333, Sigma) was added, and the slices were placed flat in a wet box and incubated at 4°C for 24 h. Thereafter, they were transferred to PBS for rinsing again, DAPI (Servicebio, Wuhan, China) was added, and incubation was performed at room temperature in the dark for 8 min. Finally, the slices were rinsed three times with PBS and sealed with Antifade Mounting Medium (Servicebio, Wuhan, China), and images were obtained on a fluorescence microscope (Nikon, Japan).

### 2.8 Plant total RNA extraction and cDNA template preparation

*A*. *paniculata* sterile seedlings at 2 months of age were divided into control and AP12 groups. After culturing for 15 days, samples were taken every 3 days and quickly frozen with liquid nitrogen. Total RNA from each sample was extracted using a Spectrum™ Total Plant RNA (Sigma, Merck, USA) Isolation Kit (Cherukupalli et al., 2016), and its quality and concentration were assessed on a B-500 ultramicro spectrophotometer (Shanghai Yuan Analysis, Shanghai, China). RNA integrity was verified by agarose gel electrophoresis (gel concentration 1.2%, 1×TAE electrophoresis buffer; 150 V, 15 min, **Supplementary Figure 1**) and then analyzed on a Peiqing JS series automatic gel imager (Shanghai Peiqing Technology, Shanghai, China). RNA with good quality and integrity was used to synthesize a cDNA template *via* a two-step method. First, 700 ng of total RNA was treated with DNase I to obtain the reverse transcription template, followed by cDNA template preparation using an Evo *M-MLV* Reverse Transcriptase Kit (Accurate Biology, China) in a 20-μl reverse transcription system consisting of the above reverse transcription template (10 μl), 5× Evo *M-MLV* RT Reaction Mix (4 μl) [Evo *M-MLV* RTase, RNase Inhibitor, dNTP, 50 μM Oligo (dT) Primer, 400 μM Random 6 mer Primers, and reaction buffer], and RNase-free water (6 μl). The PCR conditions were as follows: 37°C for 15 min, 85°C for 5 s, holding at 4°C.

### 2.9 Determination of key gene transcription level of andrographolide biosynthesis by real-time quantitative PCR

There are two pathways involved in AD biosynthesis: the MVA pathway in the cytoplasm and the MEP pathway in plastids. Ten key genes related to andrographolide biosynthesis were screened (Shen et al., 2016; Sun et al., 2019): 3-hydroxy-3-methylglutaryl-CoA synthetase (*HMGS*), 3-hydroxy-3-methylglutaryl-CoA reductase (*HMGR*), MVA kinase (*MK*), and diphospho-MVA decarboxylase (*MPDC*) in the upstream pathway of MVA; 1-deoxy-d-xylulose 5-phosphate synthetase (*DXS*), 2-c-methyl-d-erythritol 4-phosphate cytidylyltransferase (*MCT*), 4-(cytidine 5-diphosphate)-2-c-methyl-d-erythritol kinase (*CMK*), and 2-c-methyl-d-erythritol 2,4-cyclodiphosphate synthase (*MDS*) in the upstream pathway of MEP; and the key genes geranyl-geranyl pyrophosphate synthase (*GPPS*) and ent-copalyl diphosphate synthase (*CPS*), downstream of the two biosynthesis pathways. Primer Premier 6.0 software (Premier Corporation, Canada) was used to design qPCR primers with a length of 110–220 bp (**Supplementary Table 7**). The primers were synthesized by Shenggong Bioengineering Co., Ltd. (Shanghai), Among them, a pair of quantitative primers, *qActin*-F: TTCACCACTACAGCAGCG and *qActin*-R: AAGGACCTCAGGGCATCG, were designed using *A*. *paniculata* actin as the internal reference gene (Shen et al., 2016). SYBR PreMix ExTaq™ (Takara, Dalian, China) was used for qPCR on an ABI 7500 real-time polymerase chain reaction system, and the reaction conditions were as follows: predenaturation at 95°C for 30 s, followed by amplification for 45 cycles (including denaturation at 95°C for 5 s, annealing at 60°C for 30 s, and extension at 72°C for 20 s). The gene expression results were assessed by using the 2^−ΔΔCt^ method.

### 2.10 Data analysis

All data were expressed as mean ± SD, seedlings were selected at random, and all the experiments had three independent repeats; statistical significance analysis was performed by using SPSS 20.0 software (IBM, Armonk, NY, United States). For three or more groups, the measures were tested by one-way analysis of variance (ANOVA). For two groups, the measures were tested by independent samples *t*-test analysis, with *p* < 0.05 indicating a statistically significant difference. All bar charts were drawn by GraphPad Prism 8.0 software (GraphPad, San Diego, USA).

## 3 Results

### 3.1 Effects of AP12 fungal elicitor components on the growth and biomass of *A*. *paniculata*

**Figure 2:**
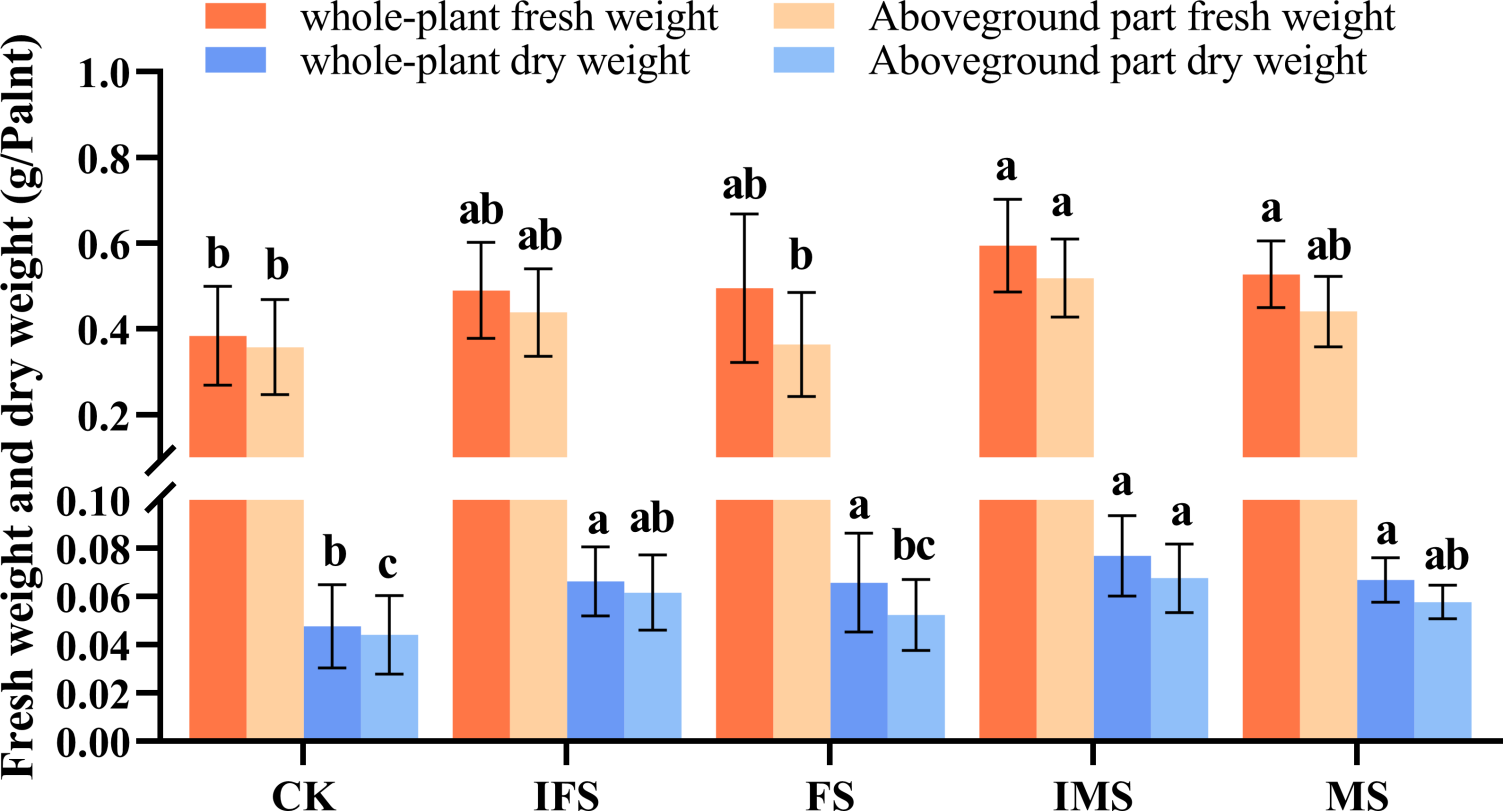
Biomass accumulation of *A*. *paniculata* irrigated with four AP12 fungal ECs for 65 days. CK, control group; IFS, inactivated fermentation solution; FS, fermentation solution; IMS, inactivated mycelium solution; MS, mycelium solution. Different lowercase letters in columns of the same color represent significant differences (*p* < 0.05).

*A*. *paniculata* plants were irrigated with the four AP12 fungal ECs and cultured in the greenhouse for 65 days. The effects of the four AP12 fungal ECs on the growth and biomass of *A*. *paniculata* were evaluated, and the results are shown in **Table 2**. Each AP12 EC treatment resulted in a significant difference (*p* < 0.05) compared with the control, and the values of three growth indicators were higher than those of the control. Among these indicators, in the MS group, plant height was 12.71 ± 1.73 cm, the number of leaves was 10.00 ± 0.00, and all indicators showed the highest values (*p* < 0.05). The IMS group showed the maximum leaf area [2.37-fold (*p* < 0.05) vs. control]. Overall, the AP12 fungal ECs positively promoted the growth of *A*. *paniculata* plants, especially the IMS elicitor. As shown in **Figure 2**, the values obtained from the AP12 fungal EC groups were all higher than those from the control in terms of the biomass of *A*. *paniculata*, especially IMS, which produced better results compared to the other treatments. The whole-plant fresh weight, whole-plant dry weight, aboveground part fresh weight, and aboveground part dry weight values reached 1.55-, 1.60-, 1.45-, and 1.55-fold of the control values, respectively. This indicated that in terms of biomass, the four AP12 fungal ECs were able to increase the fresh and dry weights of *A*. *paniculata* plants to different degrees.

**Table 2:** Growth index of *A. paniculata* irrigated with four AP12 fungal ECs for 65 days.

Groups	Plant height (cm)	Number of leaves	Leaf area (cm^2^)
CK	8.53 ± 0.85^d^	7.33 ± 1.00^c^	2.42 ± 0.84^d^
IFS	10.87 ± 2.04^bc^	9.56 ± 0.88^a^	3.14 ± 0.87^cd^
FS	10.46 ± 1.36^c^	8.44 ± 0.88^b^	3.30 ± 0.69^c^
IMS	11.92 ± 0.88^ab^	9.78 ± 0.67^a^	5.74 ± 1.02^a^
MS	12.71 ± 1.73^a^	10.00 ± 0.00^a^	4.86 ± 0.67^b^

Different lowercase letters in the same column represent p < 0.05, with significant differences.

### 3.2 Effects of AP12 fungal elicitor components on antioxidant enzymes activity of *A*. *paniculata*

**Figure 3:**
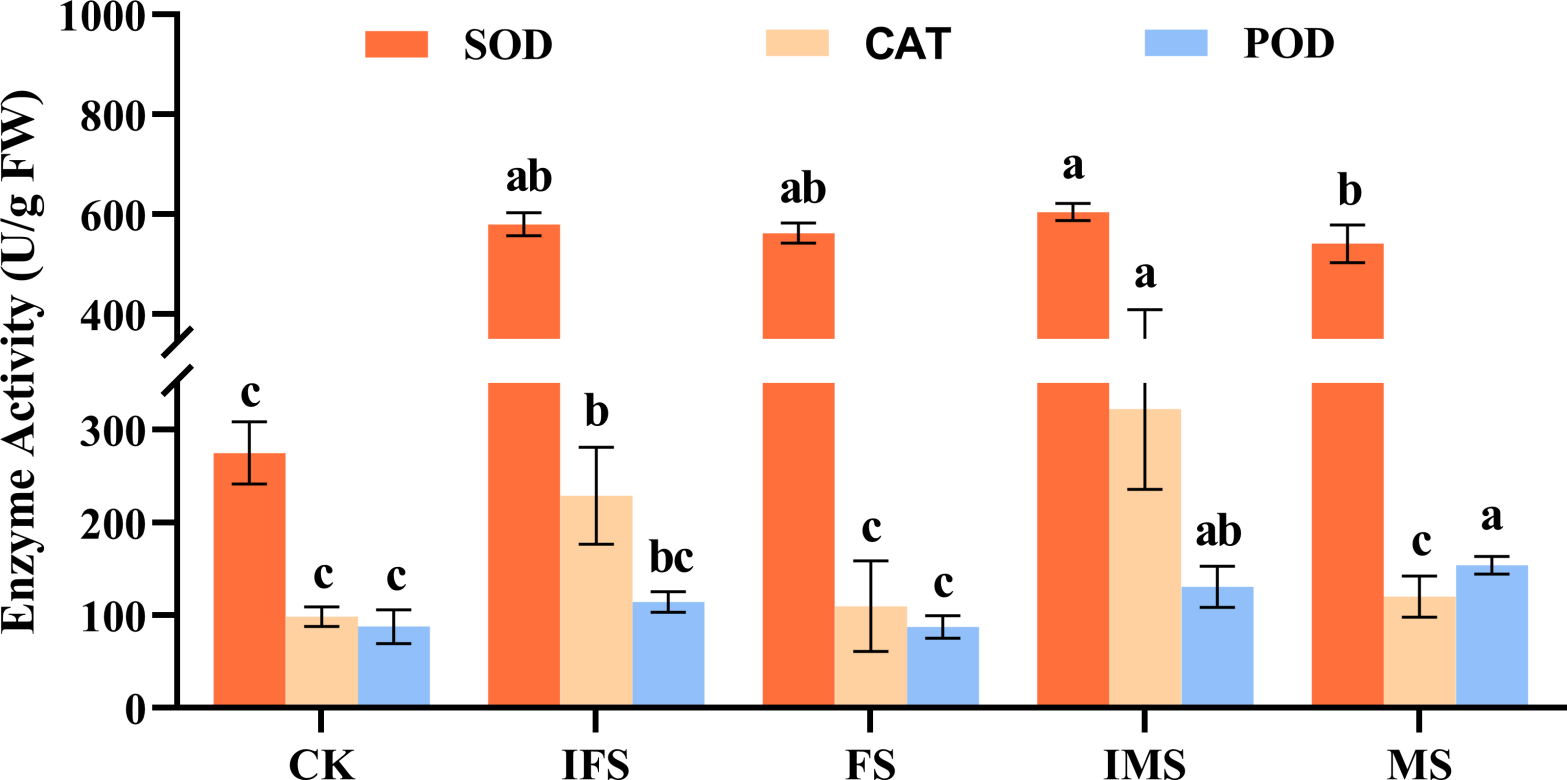
Antioxidant enzyme activities of *A*. *paniculata* irrigated with four AP12 fungal ECs for 65 days. CK, control group; IFS, inactivated fermentation solution; FS, fermentation solution; IMS, inactivated mycelium solution; MS, mycelium solution. Different lowercase letters in columns of the same color represent significant differences (*p* < 0.05).

*A. paniculata* plants were irrigated with four AP12 fungal ECs and cultured in the greenhouse for 65 days. Then, by using microplate reader detection technology, their effects on the antioxidant defense enzyme activity of *A*. *paniculata* were investigated. The results are shown in **Figure 3**. The AP12 fungal EC groups presented higher enzyme activities than the control. The SOD enzyme activity of the IMS group was increased most significantly, reaching 604.67 ± 17.25 U/g FW, which was 2.20-fold that of the control (*p* < 0.05). The increase in CAT enzyme activity was also most significant in the IMS group, reaching 322.51 ± 86.84 U/g FW, which was 3.27-fold that of the control (*p* < 0.05). The MS and IMS groups presented higher POD enzyme activity, reaching 153.99 ± 9.47 U/g FW and 130.61 ± 22.07 U/g FW, respectively, which were 1.75-fold (*p* < 0.05) and 1.49-fold that of the control.

### 3.3 Effects of AP12 fungal elicitor components on the content and yield of andrographolide compounds in *A*. *paniculata*

**Figure 4:**
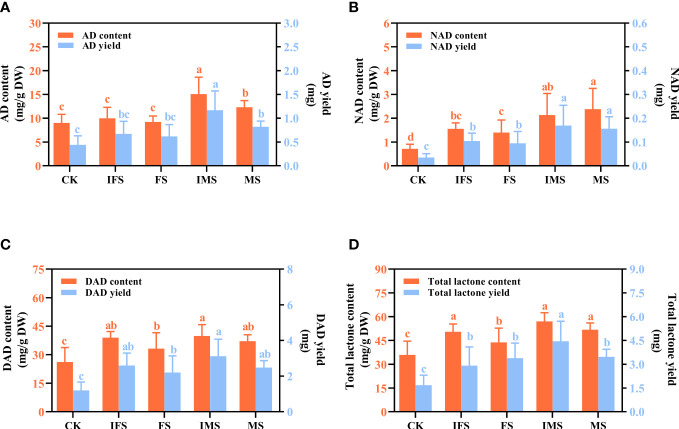
ADC content and yield of *A paniculata* irrigated with four AP12 fungal ECs for 65 days. **(A)** AD, andrographolide. **(B)** NAD, neoandrographolide. **(C)** DAD, 14-deoxyandrographolide. **(D)** Total lactone (AD+NAD+DAD). CK, control group; IFS, inactivated fermentation solution; FS, fermentation solution; IMS, inactivated mycelium solution; MS, mycelium solution. Different lowercase letters in columns of the same color represent significant differences (*p* < 0.05).

HPLC was used to investigate the effects of four AP12 fungal ECs on ADC accumulation and yield. As shown in **Figure 4**, the values obtained from the AP12 fungal ECs groups were all higher than those in the control in terms of the accumulation and yield of ADCs. Among these compounds, the IMS group produced the highest AD, DAD, and total lactone content of 15.069 ± 3.551 mg/g DW, 39.845 ± 5.962 mg/g DW, and 57.046 ± 5.496 mg/g DW, respectively, which were 1.67-fold (**Figure 4A**), 1.52-fold (**Figure 4C**), and 1.59-fold (**Figure 4D**) that of the control (*p* < 0.05). The highest NAD content was found in the MS group (**Figure 4B**), reaching 2.38 ± 0.875 mg/g DW, which was 3.35-fold that of the control (*p* < 0.05). The total lactone content and yield of the IMS group were highest, reaching 1.59-fold and 2.65-fold that of the control (*p* < 0.05), followed by the MS and FS groups. The total lactone yields were 2.01-fold and 2.06-fold that of the control (*p* < 0.05).

### 3.4 Immunocytochemical staining of the *Colletotrichum* sp. AP12-infected *A. paniculata* sterile seedlings

**Figure 5:**
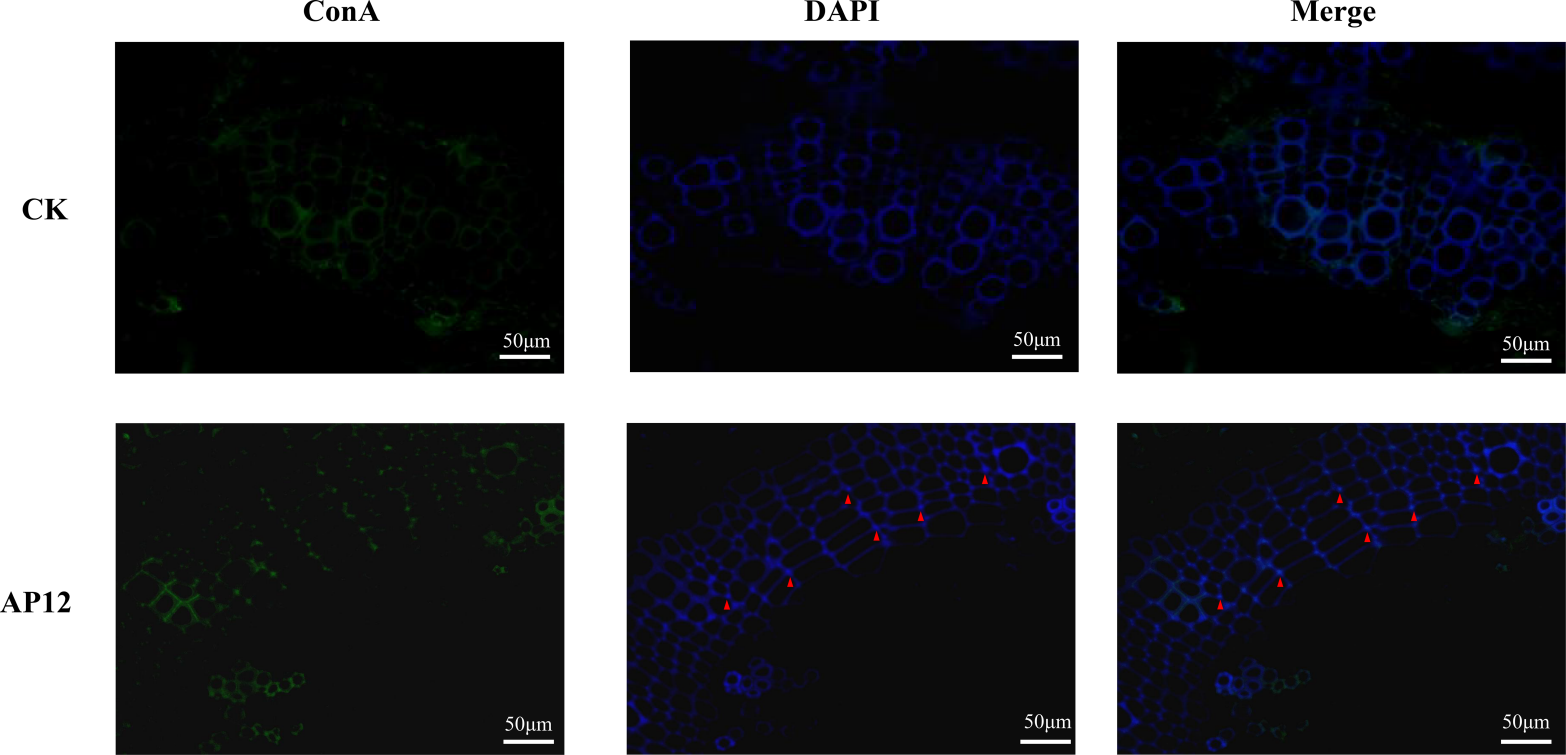
The localization of AP12-infected *A*. *paniculata* sterile seedlings after immunofluorescence staining. Green: ConA-FITC; blue: DAPI; red triangles indicated AP12 colonized in the intercellular space (magnification 400×).

In the AP12-infected sterile *A. paniculata* seedlings, immunofluorescence staining was performed to examine AP12 infection. The results are shown in **Figure 5**. AP12 mycelium was observed in the intercellular space or cell junctions (red triangle).

### 3.5 Effects of AP12-infected *A*. *paniculata* sterile seedlings on andrographolide compound content accumulation

**Figure 6:**
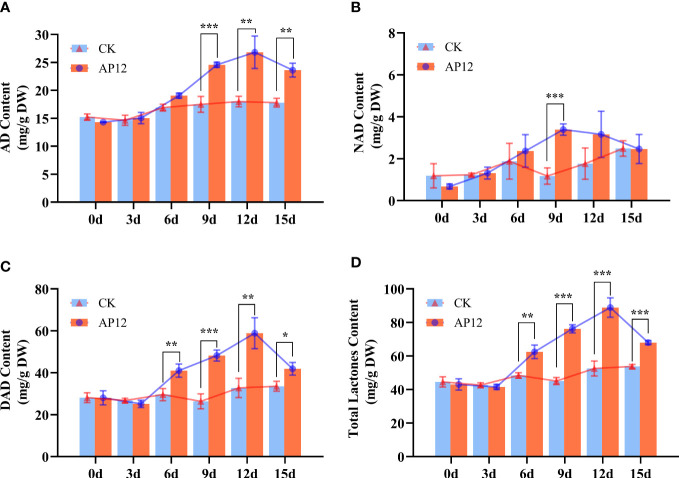
Content of AD, NAD, DAD, and total lactone content for 0, 3, 6, 9, 12, and 15 days. **(A)** AD, andrographolide. **(B)** NAD, neoandrographolide. **(C)** DAD, 14-deoxyandrographolide. **(D)** Total lactone (AD+NAD+DAD). * means *p* < 0.05, ** means *p* < 0.01, *** means *p* < 0.001, all with statistical difference.

AP12-infected sterile *A. paniculata* seedlings were cocultured for 15 days. The effect of AP12 infection on the changes in ADC content was investigated, and the results are shown in **Figure 6**. AP12 significantly increased the ADC content. On day 12, the AD content was highest (**Figure 6A**), reaching 26.815 ± 2.900 mg/g DW, which was 1.49-fold (*p* < 0.01) that of the control. On days 9 and 12, the NAD contents (**Figure 6B**) were 2.89-fold (*p* < 0.001) and 1.79-fold that of the control, respectively, and the content on day 9 was higher than that on day 12. The DAD (**Figure 6C**) and total lactone contents (**Figure 6D**) were 1.83-fold (*p* < 0.001) and 1.69-fold (*p* < 0.001) that of the control, respectively, on day 9 and 1.80-fold (*p* < 0.01) and 1.69-fold (*p* < 0.001) that of the control on day 12.

### 3.6 Transcriptional expression of key genes in the andrographolide biosynthesis pathway

**Figure 7:**
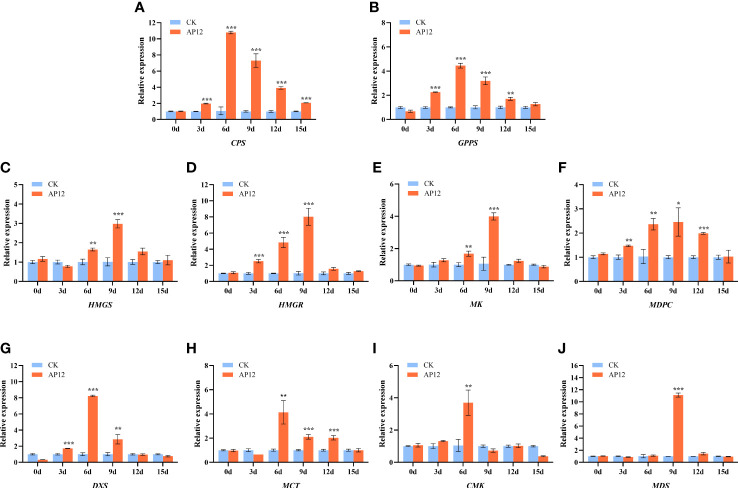
Relative expression of *HMGS*, *HMGR*, *MK*, *MPDC*, *DXS*, *MCT*, *CMK*, *MDS*, *GPPS*, and *CPS* genes for 0, 3, 6, 9, 12, and 15 days. **(A)** *CPS*, Copalyl diphosphate synthase; **(B)** *GPPS*, Geranyl pyrophosphate synthase; **(C)** *HMGS*, 3-Hydroxy-3-methylglutaryl-CoA synthase; **(D)** *HMGR*, 3-Hydroxy-3-methylglutaryl-CoA reductase; **(E)** *MK*, MVA kinase; **(F)** *MPDC*, Diphospho-MVA decarboxylase; **(G)** *DXS*, 1-Deoxy-D-xylulose 5-phosphate synthase; **(H)** *MCT*, 2-C-methyl-D-erythritol 4-phosphate cytidylyltransferase; **(I)** *CMK*, 4-(Cytidine 5-diphospho)-2-C-methyl-D-erythritol kinase; **(J)** *MDS*, 2-C-methyl-D-erythritol 2,4-cyclodiphosphate synthase. * means *p* < 0.05, ** means *p* < 0.01, *** means *p* < 0.001, all with significant difference.

To assess the transcriptional expression of key genes in the andrographolide biosynthesis pathway, we used qPCR to evaluate the relative expression changes in 10 key genes at 0, 3, 6, 9, 12, and 15 days in AP12-infected sterile *A. paniculata* seedlings. As shown in **Figure 7**, the expression levels of key genes in the andrographolide biosynthetic pathway were sequentially upregulated to different degrees. Within the upstream pathways of MVA and MEP, the genes *HMGS*, *HMGR*, *MK*, *MDPC*, *DXS*, *MCT*, *CMK*, and *MDS* were upregulated on days 3–15 after AP12 infection. The expression of the *DXS*, *MCT*, and *CMK* genes reached the highest levels on day 6, peaking at 8.22-fold (*p* < 0.001), 4.13-fold (*p* < 0.01), and 3.70-fold (*p* < 0.01) those of the control, respectively, and the *MDS* gene expression level peaked at 11.12-fold (*p* < 0.001) that of the control on day 9. Downstream of the andrographolide biosynthesis pathway, the expression levels of the *GPPS* and *CPS* genes significantly increased on day 6, reaching 4.45-fold and 10.79-fold those of the control (*p* < 0.001).

## 4 Discussion

*A*. *paniculata* has important therapeutic medicinal effects, and its market demand is greatly increasing each year (Kumar et al., 2021). However, unfavorable factors such as environmental damage and crop rotation barriers have led to unstable quality, yields, and active ingredient contents. Therefore, there is an urgent need to find new ways to breed excellent varieties (Valdiani et al., 2017; Hong et al., 2021). The endophytic fungi and their elicitors interacting with hosts (Sarkar et al., 2021) is a very effective and environmentally friendly way to improve medicinal plant quality and yields and has become a research hotspot in recent years. In particular, endophytic fungi and their elicitors have been most frequently reported to interact with *Salvia miltiorrhiza* (Ming et al., 2013; Chen et al., 2022), but there have been no reports of interactions with *A. paniculata* to date.

*Colletotrichum* sp. AP12 can produce ADCs, and we speculated that AP12 would have beneficial effects on *A*. *paniculata* plants. Therefore, in this study, AP12 was fermented to prepare four different fungal ECs (IFS, FS, IMS, and MS), which were then used to irrigate *A*. *paniculata* plants under potted conditions. The selected fungal EC types were more complete than those in recent research reports, so the interactions of the mycelium and fermentation solution of *Colletotrichum* sp. AP12 with *A*. *paniculata* could be investigated more fully. The effects of AP12 fungal ECs on *A. paniculata* growth, antioxidant defense enzyme activities, ADC contents, and yields were investigated. In this study, it was concluded that AP12 fungal ECs could promote *A*. *paniculata* growth and biomass accumulation to varying degrees (**Table 2**; **Figure 2**); in particular, IMS groups resulted in the largest leaf area and the highest biomass accumulation. Zhou et al. (2018) also reported that the endophytic fungus *Alternaria* sp. A13 could promote *S. miltiorrhiza* biomass accumulation, thereby promoting the growth of the plant. When plants are under stress, the structure of the cell membrane will be damaged (Yan et al., 2010; Singh et al., 2011), and reactive oxygen species (ROS, such as O^2−^ and H_2_O_2_) levels will increase, leading to membrane lipid peroxidation. SOD is the primary factor preventing the accumulation of various ROS in plants by converting O^2−^ to H_2_O_2_. CAT and POD work together to scavenge free radicals, converting H_2_O_2_ to H_2_O. These three antioxidant defense enzymes can work together to scavenge ROS in plants and maintain their balance, thereby reducing oxidative damage to cells. Li et al. (2022a) reported that when AM fungi and an endophytic fungus (*Piriformospora indica*) were cocultured with navel orange [*Citrus sinensis* (L.) Osb] trees, both fungi could activate the antioxidant defense system. This study evaluated the effects of four AP12 fungal ECs on the antioxidant defense enzyme activities of *A. paniculata* (**Figure 3**). The AP12 fungal EC groups presented higher SOD, CAT, and POD enzyme activities than the control, and the IMS group in particular showed significant differences from the control (*p* < 0.05), with CAT enzyme activity reaching 3.27-fold that of the control. These results indicate that AP12 can activate the antioxidant defense system of *A*. *paniculata*, improve the host’s tolerance to external stress (Hanada et al., 2010; Li et al., 2022b), and endow the AP species that interact with AP12 with excellent potential resistance, laying a foundation for the breeding of high-quality and high-resistance *A*. *paniculata* varieties. AGCs are diterpenoid lactones, which are the active ingredients exerting the therapeutic effects of *A*. *paniculata* (Islam et al., 2018). The four AP12 fungal ECs improved the ADC content and yield (**Figure 4**) according to our analysis using the HPLC method (Rafi et al., 2022); in particular, the AD content and yield in the IMS group reached 1.67-fold and 2.65-fold those of the control, respectively, and the NAD content of MS reached 3.35-fold that of the control.

Most studies in recent years of fungal elicitors’ interaction with host cultures, such as *S. miltiorrhiza* hairy roots (Ming et al., 2013), were partly due to low even non-viable survival rates after co-culture of live plants with fungal elicitors (Wang et al., 2001). In contrast, in this study, AP12 fungal ECs not only were able to coexist with *A*. *paniculata* plants in a long-term and stable manner but also promoted growth, which lays a foundation for further practical applications. The four AP12 fungal EC treatments in the *A. paniculata* cultivation application were similar in that they positively affected *A*. *paniculata* growth, defense enzyme activity, ADC content, and yield. Among the ECs, IMS, obtained by inactivating AP12 mycelia, was the most significant elicitor, indicating that inactivation treatment and mycelial part induction were particularly effective and that the mycelial component likely had more active substances beneficial to *A*. *paniculata* growth and secondary metabolism. Li et al. (2022b) reported that ADCs were detectable only in the mycelial fraction of AP12, which is further proof that the mycelia contain more active substances, may be certain growth hormones or key enzyme genes etc. (Sudha and Ravishankar, 2002). This could possibly be achieved by screening the active monomer components with an inductive effect on fermentation metabolites (Zhao et al., 2010). Zhai et al. (2018) found that *C. globosum* D38 live mycelium and the inactivated elicitor form of the mycelium could both promote *S. miltiorrhiza* growth and tanshinone biosynthesis. This study concluded that both inactivation and noninactivation treatments had positive induction effects on *A*. *paniculata*, which was consistent with the results obtained in *C. globosum* D38 cocultured with *S. miltiorrhiza* and indicated that the noninactivation treatment could also maintain a relatively stable relationship with *A*. *paniculata*, possibly because *Colletotrichum* sp. is the dominant genus in the endophytic fungi of *A*. *paniculata* and has a strong affinity with *A*. *paniculata* (Hiruma et al., 2016; Wirtz et al., 2021).

Chen et al. (2021) reported that *Mucor circinelloides* DF20 infected *S. miltiorrhiza* root sterile seedlings, and its mycelium was observed to colonize the roots by immunofluorescent chemical staining. This was because the endophytic fungal mycelium could form “infection nails” to infect plant root tips, shoot tips, and wound tissues (Yan et al., 2019). In this study, we concluded from the pot experiment that AP12 mycelium showed the best induction effect; thus, we further back-infected sterile *A. paniculata* seedling stem tips with AP12 mycelium and successfully constructed an AP12-*A*. *paniculata* infection system, based on which AP12 infection in *A*. *paniculata* stems was researched (**Figure 5**), and the AP12 mycelium was found to mainly colonize the intercellular space. This further demonstrated the beneficial interaction between AP12 and *A*. *paniculata*.

In the plant kingdom, diterpenoids are synthesized *via* both the MVA and MEP pathways. First, IPP and DMAPP are produced *via* a pathway involving a series of rate-limiting enzyme genes (*HMGS*, *HMGR*, *MK*, *MPDC*, *DXS*, *MCT*, *CMK*, and *MK*), and the *GGPS* gene product then catalyzes the formation of GGPP from one molecule of IPP and one molecule of DMAPP (Sun et al., 2019), which is a direct prerequisite for the formation of diterpenoids. CPP is produced from GGPP, which is catalyzed by the *CPS* gene product; *CPS* is the most significant key gene downstream of andrographolide synthesis (Nair and Manjula, 2020). In this study, AP12 infected *A. paniculata* sterile seedlings and further revealed the changes in the regulation of key genes in the andrographolide biosynthesis pathway by AP12 in molecular aspects, and the qPCR method (Cherukupalli et al., 2016) was used to detect the relative expression of 10 genes (**Figure 7**). The results showed that the expression of the 10 genes was slowly upregulated to varying degrees beginning on day 3 and was then downregulated to varying degrees in a sequential manner by day 9 or 12, which was consistent such that the transcription levels of the *GGPPS*, *CPS*, and *KSL* genes first increased and then decreased (Chen et al., 2021). Similarly, the content of ADCs at 0–15 days was determined by HPLC (**Figure 6**). Beginning on day 6, the ADC content was increased to varying degrees relative to the control, especially on days 9 and 12; the increase was large and began to decline on day 15. The results showed that ADC accumulation lagged behind the expression of these key genes, and studies presented previously have reported that gene expression is activated in the plant before active ingredients begin to accumulate (Ming et al., 2012). The trend of the upregulation of all 10 key genes indicated the involvement of both the MVA and MEP pathways in the response to andrographolide biosynthesis, unlike tanshinone biosynthesis, which mainly involves the MEP pathway. However, the effect was more noticeable in the early stages of colonization. The most downstream gene, *CPS*, showed a very close relationship with the andrographolide content, where the relative expression of the *CPS* gene was upregulated to synthesize more ADCs (Shen et al., 2016). In this study, the expression level of the *CPS* gene reached 10-fold that of the control. At the molecular level, this study provided ample evidence that AP12 stimulated the expression of key genes and induced the metabolic pathway of andrographolide biosynthesis, which ultimately led to the accumulation of ADCs in *A. paniculata* plants. However, it is unclear which signal transmission pathway mediates the inductions of these genes by AP12. The molecular mechanism involved therefore needs further study, possibly by using transcriptomics, metabolomics, and modern molecular technology.

## 5 Conclusion

In this study, four AP12 fungal ECs were shown to promote *A. paniculata* growth and ADC accumulation and to enhance defense enzyme activity to different degrees under greenhouse conditions. The AP12 mycelial solution induced more significant effects than the fermentation solution. In terms of the molecular mechanism involved, it was therefore shown by constructing an AP12-infected *A. paniculata* sterile seedling coculture system that AP12 can upregulate the expression levels of key genes related to andrographolide biosynthesis, which, in turn, promotes the accumulation of anti-inflammatory and antibacterial ADCs in the host. In conclusion, *Colletotrichum* sp. AP12 is an efficient inducer of the growth of *A. paniculata* and accumulation of ADCs, which is advantageous to the cultivation and application of biological bacterial fertilizer of *A. paniculata*, as well as the use of endophytic fungus, a huge microbial resource, to co-culture with *A. paniculata* plants to provide a new way to solve its quality and yield problems and develop a new idea.

## Data availability statement

The datasets presented in this study can be found in online repositories. The names of the repository/repositories and accession number(s) can be found in the article/**Supplementary Material**.

## Author contributions

DX, NL, Y-QG, and QD conceived and designed the experiments. NL and Y-QG carried out the isolation and purification of *Colletotrichum* sp. AP12 and the preparation of its elicitors. J-YZ, JH, and B-SH conducted experiments on the growth index, defense enzyme activity, ADCs content, and yield accumulation of *A*. *paniculata* by AP12 fungal elicitor irrigation. DX and Y-QG conducted experiments to construct the AP12-infected *A*. *paniculata* sterile seedling co-culture system and to explore the molecular mechanism of andrographolide biosynthesis and content accumulation. DX and J-WH analyzed the data. DX and NL wrote the draft paper. QD provided financial support for the experiments and directed the writing. All authors contributed to the article and approved the submitted version.

## Glossary

**Table:**

**Abbreviations**	**Definitions**
AD	andrographolide
ADCs	andrographolide compounds
*A. paniculata*	*Andrographis paniculata* (Burm. f.) Nees
BSA	bovine serum albumin
CAT	catalase
*CMK*	4-(cytidine 5-diphosphate)-2-C-methyl-D-erythritol kinase
*CPS*	ent-copalyl diphosphate synthase
DAD	14-deoxyandrographolide
DAPI	4’,6-diamidino-2-phenylindole
DMAPP	dimethyl allyl diphosphate
*DXS*	1-deoxy-D- xylulose 5-phosphate synthetase
ECs	elicitor components
EDTA	ethylene diamine tetraacetic acid
FITC	fluorescein isothiocyanate
FS	fermentation solution
*GPPS*	geranyl pyrophosphate synthase
*HMGR*	3-hydroxy-3-methylglutaryl-CoA reductase
*HMGS*	3-hydroxy-3-methylglutaryl-CoA synthetase
IFS	inactivated fermentation solution
IMS	inactivated mycelium solution
IPP	isopentenyl diphosphate
*MCT*	2-C-methyl-D-erythritol 4-phosphate cytidylyltransferase
*MDS*	2-C-methyl-D-erythritol 2,4-cyclodiphosphate synthase
MEP	2-C-methyl-D-erythritol-4-phosphate pathway
*MK*	MVA kinase
*MPDC*	diphospho-MVA decarboxylase
MS	mycelium solution
MVA	mevalonate pathway
NAD	neoandrographolide
PBS	phosphate buffer saline
PDA	potato dextrose agar
PDB	potato dextrose broth
POD	peroxidase
ROS	reactive oxygen species
SOD	superoxide dismutase
