# Supplementary material for: Endophytic fungus Colletotrichum sp. AP12 promotes growth physiology and andrographolide biosynthesis in Andrographis paniculata (Burm. f.) Nees
Source: Front Plant Sci. 2023 Jul 4;14:1166803. doi: 10.3389/fpls.2023.1166803 (PMC10353853; doi:10.3389/fpls.2023.1166803)
Supplement: Supplementary file 1 [file DataSheet_1.docx]

Supplementary Material

# Supplementary Tables

# Supplementary Table 1 Biomass accumulation of *A. paniculata* irrigated with four AP12 fungal ECs for 65d*s* (n±x).

| Weight (g/Plant) | | | | |
| --- | --- | --- | --- | --- |
| Groups | whole-plant fresh | whole-plant dry | aboveground part fresh | aboveground part dry |
| CK | 0.384±0.115^b^ | 0.048±0.017^b^ | 0.358±0.111^b^ | 0.044±0.016^b^ |
| IFS | 0.490±0.112^ab^ | 0.066±0.014^ab^ | 0.439±0.102^ab^ | 0.062±0.016^a^ |
| FS | 0.496±0.173^ab^ | 0.066±0.021^ab^ | 0.364±0.121^b^ | 0.053±0.015^ab^ |
| IMS | 0.594±0.108^a^ | 0.077±0.017^a^ | 0.519±0.091^a^ | 0.068±0.014^a^ |
| MS | 0.528±0.077^ab^ | 0.067±0.009^ab^ | 0.441±0.082^b^ | 0.058±0.007^ab^ |

Different lowercase letters in the same column represent *p* < 0.05, with significant differences.

# Supplementary Table 2 Antioxidant enzyme activities of *A. paniculata* irrigated with four AP12 fungal ECs for 65 d (n±x).

| Groups | Enzyme activity (U/g FW) | | |
| --- | --- | --- | --- |
|  | SOD | CAT | POD |
| CK | 275.03±33.59^c^ | 98.62±10.49^c^ | 87.79±18.21^c^ |
| IFS | 580.18±20.07^ab^ | 228.67±52.26^b^ | 114.29±10.92^bc^ |
| FS | 562.31±23.05^ab^ | 109.77±48.9^c^ | 87.27±12.24^c^ |
| IMS | 604.67±17.25^a^ | 322.51±86.84^a^ | 130.61±22.07^ab^ |
| MS | 541.42±37.98^b^ | 120.13±22.18^c^ | 153.99±9.47^a^ |

Different lowercase letters in the same column represent *p* < 0.05, with significant differences.

# Supplementary Table 3 ADCs content of *A. paniculata* irrigated with four AP12 fungal ECs for 65 d (n±x).

| Groups | Content (mg/g DW) | | | |
| --- | --- | --- | --- | --- |
|  | AD | NAD | DAD | Total lactone (AD+NAD+DAD) |
| CK | 9.001±1.801^c^ | 0.711±0.198^d^ | 26.174±7.57^c^ | 35.887±8.648^c^ |
| IFS | 9.981±2.305^c^ | 1.557±0.242^bc^ | 38.984±3.198^ab^ | 50.522±4.865^a^ |
| FS | 9.211±1.23^c^ | 1.399±0.525^c^ | 33.201±8.333b | 43.811±8.930^b^ |
| IMS | 15.069±3.551^a^ | 2.131±0.91^ab^ | 39.845±5.962^a^ | 57.046±5.496^a^ |
| MS | 12.299±1.41^b^ | 2.38±0.875^a^ | 37.121±3.385^ab^ | 51.800±4.264^a^ |

Different lowercase letters in the same column represent *p* < 0.05, with significant differences.

# Supplementary Table 4 ADCs yield of *A. paniculata* irrigated with four AP12 fungal ECs for 65 d (n±x).

| Groups | Yield (mg DW) | | | |
| --- | --- | --- | --- | --- |
|  | AD | NAD | DAD | Total lactone |
| CK | 0.44±0.192^d^ | 0.035±0.016^d^ | 1.202±0.469^c^ | 1.677±0.636^c^ |
| IFS | 0.618±0.248^bc^ | 0.105±0.033^bc^ | 2.599±0.691^ab^ | 2.918±1.168^b^ |
| FS | 0.673±0.26b^c^ | 0.095±0.05^c^ | 2.206±0.93^b^ | 3.377±0.955^b^ |
| IMS | 1.167±0.407^a^ | 0.169±0.085^a^ | 3.111±0.957^a^ | 4.447±1.264^a^ |
| MS | 0.82±0.119^b^ | 0.156±0.05^ab^ | 2.484±0.388^ab^ | 3.460±0.481^b^ |

Different lowercase letters in the same column represent *p* < 0.05, with significant differences.

# Supplementary Table 5 Content of AD, NAD, DAD and total lactone content for 0, 3, 6, 9, 12, 15 d (n±x).

| Groups | AD Content (mg/g DW) | | | | | |
| --- | --- | --- | --- | --- | --- | --- |
|  | 0 d | 3 d | 6 d | 9 d | 12 d | 15 d |
| CK | 15.235±0.567 | 14.639±0.92 | 16.902±0.618 | 17.497±1.424 | 18.009±0.947 | 17.808±0.794 |
| AP12 | 14.314±0.087 | 15.057±1.001 | 19.057±0.474** | 24.584±0.495*** | 26.815±2.900** | 23.62±1.233** |
|  |  |  |  |  |  |  |
| Groups | NAD Content (mg/g DW) | | | | | |
|  | 0 d | 3 d | 6 d | 9 d | 12 d | 15 d |
| CK | 1.188±0.578 | 1.231±0.084 | 1.876±0.855 | 1.174±0.389 | 1.761±0.751 | 2.489±0.368 |
| AP12 | 0.672±0.127 | 1.31±0.282 | 2.371±0.782 | 3.392±0.270*** | 3.158±1.103 | 2.459±0.696 |
|  |  |  |  |  |  |  |
| Groups | DAD Content (mg/g DW) | | | | | |
|  | 0 d | 3 d | 6 d | 9 d | 12 d | 15 d |
| CK | 28.097±2.382 | 26.78±1.039 | 29.603±2.904 | 26.372±3.585 | 32.721±4.598 | 33.522±2.358 |
| AP12 | 28.077±3.371 | 25.172±1.734 | 40.979±3.197** | 48.182±2.623*** | 58.908±7.412** | 41.885±3.028* |
|  |  |  |  |  |  |  |
| Groups | Total lactone Content (mg/g DW) | | | | | |
|  | 0 d | 3 d | 6 d | 9 d | 12 d | 15 d |
| CK | 44.52±3.007 | 42.649±1.42 | 48.382±1.643 | 45.043±2.105 | 52.491±4.471 | 53.819±1.243 |
| AP12 | 43.063±3.386 | 41.539±1.533 | 62.407±4.026** | 76.157±2.468*** | 88.881±5.793*** | 67.963±1.147*** |

* means *p*<0.05, ** means *p*<0.01, *** means *p*<0.001, all with statistical difference.

# Supplementary Table 6 Relative expression of *HMGS, HMGR, MK, MPDC DXS, MCT, CMK, MDS GPPS* and *CPS* genes for 0, 3, 6, 9, 12, 15 d (n±x).

| *HMGS* | 0 d | 3 d | 6 d | 9 d | 12 d | 15 d |
| --- | --- | --- | --- | --- | --- | --- |
| CK | 1.00±0.09 | 1.00±0.10* | 1.00±0.15 | 1.00±0.21 | 1.00±0.13 | 1.00±0.08 |
| AP12 | 1.16±0.12 | 0.78±0.06 | 1.65±0.08** | 2.98±0.21*** | 1.55±0.17* | 1.10±0.25 |

| *HMGR* | 0 d | 3 d | 6 d | 9 d | 12 d | 15 d |
| --- | --- | --- | --- | --- | --- | --- |
| CK | 1.00±0.03 | 1.00±0.12 | 1.00±0.03 | 1.00±0.23 | 1.00±0.18 | 1.00±0.12 |
| AP12 | 1.08±0.12 | 2.52±0.21*** | 4.84±0.63*** | 8.02±1.05*** | 1.57±0.18* | 1.27±0.04* |

| *MK* | 0 d | 3 d | 6 d | 9 d | 12 d | 15 d |
| --- | --- | --- | --- | --- | --- | --- |
| CK | 1.00±0.05 | 1.00±0.15 | 1.00±0.13 | 1.00±0.41 | 1.00±0.03 | 1.00±0.04 |
| AP12 | 0.95±0.03 | 1.28±0.1 | 1.68±0.16** | 4.00±0.23*** | 1.24±0.11* | 0.88±0.08 |

| *MDPC* | 0 d | 3 d | 6 d | 9 d | 12 d | 15 d |
| --- | --- | --- | --- | --- | --- | --- |
| CK | 1.00±0.06 | 1.00±0.09 | 1.00±0.30 | 1.00±0.06 | 1.00±0.06 | 1.00±0.09 |
| AP12 | 1.14±0.04* | 1.47±0.03** | 2.37±0.24** | 2.46±0.59* | 1.99±0.04*** | 1.03±0.26 |

| *DXS* | 0 d | 3 d | 6 d | 9 d | 12 d | 15 d |
| --- | --- | --- | --- | --- | --- | --- |
| CK | 1.00±0.08 | 1.00±0.10 | 1.00±0.17 | 1.00±0.20 | 1.00±0.09 | 1.00±0.07 |
| AP12 | 0.32±0.01 | 1.73±0.03*** | 8.22±0.10*** | 2.85±0.58** | 0.96±0.09 | 0.76±0.10 |

| *MCT* | 0 d | 3 d | 6 d | 9 d | 12 d | 15 d |
| --- | --- | --- | --- | --- | --- | --- |
| CK | 1.00±0.05 | 1.00±0.11 | 1.00±0.09 | 1.00±0.05 | 1.00±0.08 | 1.00±0.09 |
| AP12 | 0.97±0.10 | 0.65±0.01 | 4.13±0.97** | 2.10±0.19*** | 2.03±0.18*** | 1.00±0.15 |

| *CMK* | 0 d | 3 d | 6 d | 9 d | 12 d | 15 d |
| --- | --- | --- | --- | --- | --- | --- |
| CK | 1.00±0.03 | 1.00±0.15 | 1.00±0.37 | 1.00±0.07 | 1.00±0.07 | 1.00±0.05 |
| AP12 | 1.06±0.12 | 1.32±0.04* | 3.70±0.78** | 0.73±0.10 | 1.02±0.11 | 0.38±0.03 |

| *MDS* | 0 d | 3 d | 6 d | 9 d | 12 d | 15 d |
| --- | --- | --- | --- | --- | --- | --- |
| CK | 1.00±0.05 | 1.00±0.06 | 1.03±0.27 | 1.00±0.01 | 1.00±0.01 | 1.00±0.05 |
| AP12 | 1.04±0.03 | 0.87±0.04 | 1.10±0.12 | 11.12±0.34*** | 1.44±0.21* | 0.94±0.04 |

| *GPPS* | 0 d | 3 d | 6 d | 9 d | 12 d | 15 d |
| --- | --- | --- | --- | --- | --- | --- |
| CK | 1.00±0.08 | 1.00±0.07 | 1.00±0.04 | 1.01±0.13 | 1.00±0.10 | 1.00±0.09 |
| AP12 | 0.68±0.10 | 2.26±0.02*** | 4.45±0.20*** | 3.19±0.32*** | 1.71±0.13** | 1.27±0.14 |

| *CPS* | 0 d | 3 d | 6 d | 9 d | 12 d | 15 d |
| --- | --- | --- | --- | --- | --- | --- |
| C | 1.00±0.05 | 1.00±0.03 | 1.00±0.48 | 1.00±0.10 | 1.00±0.11 | 1.00±0.05 |
| AP12 | 1.01±0.04 | 1.98±0.05*** | 10.79±0.16*** | 7.31±0.84*** | 3.91±0.17*** | 2.07±0.01*** |

* means *p*<0.05, ** means *p*<0.01, *** means *p*<0.001, all with statistical difference.

# Supplementary Table 7 qPCR primer sequences and amplified fragment size.

| Gene Name | Primer Sequences (5’-3’) |
| --- | --- |
| *HMGS*-F | TGCAGCCGTCCACACTCAT |
| *HMGS*-R | CGGACAGGTCGGTGTTCTTG |
| *HMGR*-F | TTCGTGATGGAATGACCAGA |
| *HMGR*-R | TTGCAAATCTGCTTGACCTG |
| *MK*-F | CGGCATTGACAACACAGTAAGC |
| *MK*-R | TTTCTGACACACCTGCAACCAA |
| *MPDC*-F | GCGACGAGGACAGGATTCTTC |
| *MPDC*-R | CTCCTTGCCGTTGAGCCAAA |
| *DXS*-F | GGCAGACGGACCTACACATT |
| *DXS*-R | GTGGCAACCATGTGAAACAG |
| *MCT*-F | GGCGTATTGGTGCTGCTGTT |
| *MCT*-R | AGGCTGGCTTAATAACCTGTGG |
| *CMK*-F | CAATGCTGCCACTGCTCTGT |
| *CMK*-R | TTCGACTACCTCGCCTCTTCC |
| *MDC*-F | GTTATGCTGCTCCGCTTGCTA |
| *MDC*-R | GACGACAACGACGAAGGTCTC |
| *GPPS*-F | GTTGTCAAGGTGCATTGGTG |
| *GPPS*-R | GCTCCACTTCCGACTCTGTC |
| *CPS*-F | GGAGAAATACAACAAACACTGG |
| *CPS*-R | CGTCCATTGATCTCAGCATTG |

# Supplementary Figures

#
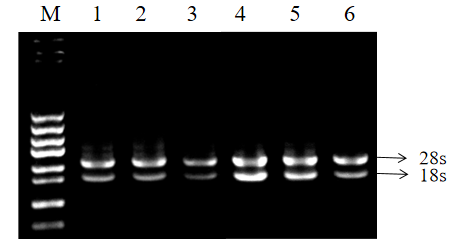
Supplementary Figures 1

Total RNA electrophoresis bands of *A. paniculata*: M represents 5Kb DNA maker, 1-3 represent CK samples, and 4-6 represent AP12 samples, respectively.
